# Significance of HLA Haplotypes in Two Patients with Subacute Thyroiditis Triggered by mRNA-Based COVID-19 Vaccine

**DOI:** 10.3390/vaccines10020280

**Published:** 2022-02-11

**Authors:** Magdalena Stasiak, Katarzyna Zawadzka-Starczewska, Andrzej Lewiński

**Affiliations:** 1 Department of Endocrinology and Metabolic Diseases, Polish Mother’s Memorial Hospital—Research Institute, 281/289 Rzgowska St., 93-338 Lodz, Poland; mstasiak33@gmail.com (M.S.); kasiula891@op.pl (K.Z.-S.); 2 Department of Endocrinology and Metabolic Diseases, Medical University of Lodz, 281/289 Rzgowska St., 93-338 Lodz, Poland

**Keywords:** subacute thyroiditis, COVID-19 vaccine, SARS-CoV-2, HLA

## Abstract

Subacute thyroiditis (SAT) can be triggered by several viral factors in genetically predisposed individuals. In the case of COVID-19, SAT can be induced by SARS-CoV-2 infection as well as COVID-19 vaccination. The aim of this study was to present two cases of SAT triggered by mRNA-based COVID-19 vaccines, with special attention paid to the possible significance of HLA-related SAT susceptibility. In our patients, a strong similarity of HLA profiles with regard not only to SAT high-risk alleles but also to other SAT-unrelated ones was observed. The rare phenomenon of SAT occurrence after COVID-19 vaccination can be HLA-dependent and related to a co-presence of HLA-B*35:03 and -C*04:01. Taking into account the similarity of HLA profiles in both our patients, the co-presence of other alleles, such as HLA-A*03:01, *-DQA1:01*, DQB1*05:01 as well as some of *HLA-DRB1*, can also play a role. This hypothesis is strongly consistent with autoimmune/inflammatory syndrome induced by adjuvants (ASIA) being the postulated mechanism of this post-vaccine reaction, as ASIA-related immune reactions are directly associated with HLA-based genetic susceptibility. Further research is necessary to confirm these findings.

## 1. Introduction

Subacute thyroiditis (SAT) (also called de Quervain’s disease) is a destructive inflammatory thyroid disease, which is usually induced by viral factors in individuals with genetic predisposition [1,2]. The genetic susceptibility to SAT is associated with a presence of specific HLA alleles including HLA-B*35, HLA-B*18:01, -DRB1*01:01, and -C*04:01 [2]. Patients with SAT usually complain of anterior neck pain radiating to the mandible, ear and upper chest [1], although a painless course has recently been more often described [3,4]. Fever, fatigue, malaise and symptoms of thyrotoxicosis are also common. Laboratory tests reveal a highly accelerated erythrocyte sedimentation rate (ESR), commonly with elevated serum C-reactive protein (CRP) and laboratory signs of thyrotoxicosis. Ultrasound (US) characteristically reveals hypoechoic areas with blurred margins and reduced vascularization [1,4].

For the last two years, the SARS-CoV-2 pandemic has spread around the world and COVID-19 has become the most frequent infectious disease ever. SARS-CoV-2 was demonstrated to be the first coronavirus associated with SAT development [4,5,6] and the virus has been included in the list of viral SAT triggers [7].

Governments all over the world undertook a massive vaccination campaign in an attempt to protect populations against COVID-19. Up to January 2022, 9.79 billion doses have been administered globally [8]. Several reports of SAT after various types of COVID-19 vaccines have already been published [9,10,11,12,13,14,15,16,17,18,19]. Rare cases of SAT triggered by other vaccinations have been described before, mainly in regard to influenza or hepatitis B vaccines [20,21]. However, taking into account a tremendous number of people vaccinated against COVID-19 worldwide, it remains unclear why SAT occurs in such a small percentage of them and what are the potential risk factors. The correlation between HLA-related genetic susceptibility and clinical course of SAT induced either by SARS-CoV-2 infection or other factors has recently been demonstrated [22,23,24,25].

The aim of this study is to present two cases of SAT triggered by COVID-19 vaccinations with special attention paid to the possible significance of HLA-related SAT susceptibility.

## 2. Case Presentation

### 2.1. Case 1

On 4 February 2021, a 51-year-old man received the second dose of the Pfizer-BioNTech mRNA-based COVID-19 vaccine. For several days after vaccination he suffered from tachyarrhythmias, elevated body temperature (never exceeding 38 °C) and excessive sweating. At the end of February, right side neck pain occurred and antibiotic therapy was introduced by a general practitioner due to the significant pain severity. Not only did the pain not subside but the other side of the neck also began to be affected. Therefore, the patient was referred to our Department for further management. On the basis of clinical presentation, laboratory results, ultrasound images and a fine needle aspiration biopsy (FNAB) (Table 1), the patient was finally diagnosed with SAT and a treatment with prednisone was started, with a rapid reduction in symptoms and gradual normalization of inflammatory parameters. The laboratory tests showed significantly accelerated ESR and features of thyrotoxicosis (Table 1). The patient’s HLA profile revealed genetic susceptibility with the heterozygous presence of two high-risk alleles (Table 2).

### 2.2. Case 2

A 45-year-old male patient was diagnosed with SAT in December 2020. At the time of diagnosis, his PCR swab test for SARS-CoV-2 infection was negative but he had reported an upper respiratory tract infection a few weeks earlier (no PCR test had been performed that time). He was treated with prednisone with rapid pain relief and subsequent improvement in laboratory results. The doses of prednisone were gradually reduced and the therapy was terminated with complete resolution of clinical, laboratory and sonographic SAT symptoms.

On 19 August 2021, the patient received the second dose of the Pfizer-BioNTech COVID-19 vaccine. After three weeks, SAT symptoms recurred with slightly less severe neck pain than during the first SAT episode. Laboratory tests and sonographic images were consistent with SAT (Table 1). Recurrence of SAT was diagnosed and treatment with prednisone was applied with slow dose reduction. Complete resolution of US lesions was achieved after three months and the treatment was withdrawn.

The patient’s HLA profile revealed genetic susceptibility to SAT with the heterozygous presence of three high-risk alleles (Table 2).

## 3. Materials and Methods

### 3.1. SAT Diagnosis Procedures

In the diagnostic process, we followed the criteria recently proposed by our research team [4]. Therefore, the diagnosis was based on elevation of ESR (or at least CRP) plus hypoechoic area/areas with blurred margin and decreased vascularization in US plus FNAB confirmation of SAT in doubtful cases plus at least one of the following: hard thyroid swelling and/or pain and tenderness of the thyroid gland/lobe and/or elevation of serum FT4 and suppression of TSH.

DNA was extracted from peripheral blood. *HLA-A*, *-B*, *-C*, *-DQB1* and *-DRB1* were genotyped using a next-generation sequencing method on an Illumina platform (Illumina, San Diego, CA, USA). Sequencing-based HLA typing of all the HLA genes was performed in a 96-well format within a semi-automated workflow by using MiaFora Flex5 typing kits (Immucor, Peachtree Konars, GA, USA). The sequencing results were analyzed by MiaFora NGS software. Data were considered sufficient for the analysis whenever the coverage reached 40 and number of cReads exceeded 50,000.

Serum concentrations of TSH, FT3 and FT4 were measured by electrochemiluminescence immunoassay (ECLIA) using a Cobas e601 analyzer (Roche Diagnostics, Indianapolis, IN, USA). ESR was determined with Ves-Matic Cube 30 (Diesse, Monteriggioni, Tuscany, Italy) and CRP was analyzed by a VITROS^®^4600 Chemistry System (Ortho Clinical Diagnostics, Raritan, NJ, USA).

US imaging was performed using a 7–14 MHz linear transducer (Toshiba Aplio XG; Toshiba, Japan). FNAB was performed with a 23-gauge needle. The presence of multinucleated giant cells together with mononucleated macrophages and follicular epithelial cells with acute and chronic inflammatory dirty background (cellular debris and mixed inflammatory cells) was considered a cytology result typical for SAT.

### 3.2. Consent Procedures

The patients gave informed written consents for all the procedures performed and signed a consent to the publication of their medical data. The consent form was accepted by the Bioethics Committee of the Polish Mother’s Memorial Hospital–Research Institute, Lodz, Poland (approval code 108/2018).

## 4. Discussion

In response to the worldwide spread of the COVID-19 pandemic, several vaccine preparations have been developed, including mRNA-based SARS-CoV-2 vaccines (BNT162b2–Pfizer-BioNTech and mRNA-1273–Moderna), an inactivated SARS-CoV-2 vaccine (CoronaVac–Sinovac Life Sciences), and viral vector vaccines (ChAdOx1 nCoV-19–Oxford-AstraZeneca and Ad26.COV2.S–Janssen–Johnson & Johnson). Several cases of SAT triggered by different types of COVID-19 vaccines have already been described [9,10,11,12,13,14,15,16,17,18,19]. However, taking into account the number of 9.79 billion doses of COVID-19 vaccines administered worldwide, SAT appears to be a rare side effect of vaccination. Thus, there is a need to explain this phenomenon. One of the possible mechanisms of vaccine-related SAT induction is an autoimmune/inflammatory syndrome induced by adjuvants (ASIA) [9], which occurs in genetically predisposed individuals [26].

Taking into account the significance of HLA-related susceptibility for SAT, we aimed to present HLA haplotypes of the two patients with SAT induced by mRNA-based COVID-19 vaccines, including one de novo case and one recurrence. In both of our patients, heterozygous co-presence of the two high-risk alleles HLA-B*35:03 and -C*04:01 was detected. HLA-C*04:01 is in linkage disequilibrium with HLA-B*35:03, i.e., these two alleles can more commonly occur together due to the close location of their loci [27]. Therefore, these two alleles cannot be considered as independent risk markers, although a presence of any of them increases SAT susceptibility [2]. It is difficult to speculate now whether such co-presence of HLA alleles can be associated with increased risk of SAT induced by COVID-19 vaccination because HLA genotyping results are not available for any other group of such patients. Obviously, a larger group of similar cases is necessary to compare their HLA haplotypes in order to draw reliable conclusions and our report should be considered as an initial observation. However, the co-presence of HLA-B*35:03 and -C*04:01 in both of the patients can play an important role in the process of SAT development after COVID-19 vaccination.

As has been underlined before, ASIA occurs in genetically predisposed individuals and it is related to autoimmune and/or inflammatory reactions that are HLA dependent [28]. The presence of specific HLA is one of the diagnostic criteria of ASIA [26]. Other criteria include: exposure to external stimuli (adjuvants), typical clinical manifestation and a presence of specific autoantibodies or antibodies to the adjuvant [26]. Thus, SAT development due to ASIA in patients with HLA-related predisposition is highly probable. Additionally, the presence of *HLA-DRB1* was postulated as a risk factor for ASIA triggered by different adjuvants [26]. In one of our patients, HLA-DRB1*01:01 is also present and should be mainly considered as an SAT high-risk factor. However, a potential enhancing influence on ASIA development cannot be excluded. Another *HLA-DRB1*-group allele is also present in the other patient i.e., -DRB1*04:01 (Table 2). Although both of our patients had very similar HLA profiles related to a high risk of SAT development, unexpected HLA concordance in regard to other alleles should be underlined. Except for SAT-typical HLA-B*35:03 and -C*04:01, both patients had a heterozygous presence of HLA-A*01:01, -A*03:01, *-DQA1:01*, DQB1*05:01 (Table 2). HLA-A*01:01 is a the most common HLA-A allele in Caucasian populations [29,30], but such a high similarity of other HLA alleles should not be considered random. One should suspect that such a similarity can play a role in the development of ASIA, and in the presence of SAT high-risk alleles, it can result in SAT triggered by COVID-19 vaccination.

Another unexpected similarity is gender. SAT is more commonly present in women, and females account for 75–80% of all SAT patients [1]. However, both of our patients were men. Both also had a similar time lag between the vaccination and the onset of SAT symptoms (three weeks) and both had SAT triggered by the second dose of the same vaccine (BNT162b2–Pfizer-BioNTech), which obviously carried the same adjuvants. It remains unclear why the reaction was only after the second dose, although a mechanism related to immune memory and immunization with already known pathogens can be considered [31].

Our present speculations can be supported by earlier observations reported by our research team. Specific sets of SAT high-risk alleles were found to be correlated with different SAT course [22,23,24,25] and the co-presence of other alleles was postulated as a potential factor that can additionally influence SAT course [24]. It has been demonstrated that the presence of HLA-B*18:01 as a single high-risk allele can be associated with atypical US patterns with SAT lesions resembling a large thyroid tumor [22]. Additionally, (the) co-presence of HLA-B*35:01 and -B*18:01 was found to increase the recurrence rate approximately nine times, as compared to other SAT high-risk profiles [23]. Moreover, a modified course of SAT in three siblings with coexistence of an HLA-related predisposition for SAT and Graves’ disease (GD) was also reported as potentially HLA-dependent [24]. In these patients, GD occurred only in one of them, while the other two suffered from much more severe thyrotoxicosis in the course of SAT. The differences in the clinical course were completely concordant with the differences in the HLA profiles, and the co-occurrence of HLA-DRB1*15:01 and/or -B*07:02, possibly together with the lack of HLA-A*01:01 and -B*41:01 seemed to be a key factor protecting against the development of GD, as well as against the recurrent SAT course and steroid dependence [24]. Very recently, the association of HLA-B*35 homozygosity with the rapid onset of SAT induced by SARS-CoV-2 infection was also postulated [25]. Therefore, the significance of specific HLA profiles covering a particular set of alleles from the high-risk group should also be considered as highly probable in the case of SAT triggered by COVID-19 vaccination.

We believe that the present report sheds new light on the potentially significant role of HLA-related susceptibility in the development of SAT after COVID-19 vaccination. Our initial observations require further confirmation in a larger group of patients with full HLA genotyping results available.

## 5. Conclusions

A rare phenomenon of SAT occurrence after COVID-19 vaccination may be HLA-dependent and related to the specific HLA profile covering the simultaneous presence of HLA-B*35:03 and -C*04:01. Taking into account the similarity of HLA profiles in both our patients, the co-presence of other alleles, such as HLA-A*03:01, *-DQA1:01*, DQB1*05:01 and some of the *HLA-DRB1*-group alleles, can also play a role. This hypothesis is strongly consistent with the postulated mechanism of the post-vaccine reaction being ASIA, as ASIA-related immune reactions are directly associated with HLA-based genetic susceptibility. Further research is necessary to confirm these findings.

## Figures and Tables

**Table 1:** The most important results of the patients’ laboratory tests and clinical findings at the time of SAT diagnosis.

Analyzed Parameter (Reference Range)	Case 1	Case 2
TSH (0.27–4.2 mIU/L)	<0.005	0.339
FT3 (2–4.4 pg/mL)	8.31	5.84
FT4 (0.93–1.7 ng/dL)	3.21	2.02
aTPO (<34 IU/mL)	11.7	9.8
aTg (<115 IU/mL)	363.1	16.8
TRAb (<1.7 IU/L)	<0.8	<0.8
ESR (<10 mm/h))	119	48
CRP (<1 mg/dL)	15.79	36.68
Neck pain	yes	yes
Body temperature	38.2 °C	36.6 °C
Heart rate (beats/min)/rhythm	102/sinus rhythm	85/sinus rhythm
Blood pressure (mmHg)	138/88	130/90
Sonographic pattern	Hypoechoic, irregular areas of both thyroid lobes	Hypoechoic, irregular area 22 × 24 × 34 mm in the right thyroid lobe
Time lag from COVID-19 vaccination	3 weeks after the 2nd dose	3 weeks after the 2nd dose
Type of vaccine	mRNA-based	mRNA-based

Abbreviations: aTg, thyroglobulin antibodies; aTPO, thyroid peroxidase antibodies; CRP, C-reactive protein; ESR, erythrocyte sedimentation rate; FT3, free triiodothyronine; FT4, free thyroxine; TRAb, thyrotropin receptor antibodies; TSH, thyrotropin.

**Table 2:** HLA genotyping results of the presented patients.

Case No	Gender	HLA- A *	HLA- A *	HLA- B *	HLA- B *	HLA- C *	HLA- C *	HLA- DRB1 *	HLA- DRB1 *	HLA- DQA1 *	HLA- DQA1 *	HLA- DQB1 *	HLA- DQB1 *
**1**	Male	**01:01**	**03:01**	**35:03 ^1^**	44:02	02:02	**04:01 ^1^**	04:01	16:01	**01:01**	01:02	**05:01**	05:02
**2**	Male	**01:01**	**03:01**	**35:03 ^1^**	08:01	07:01	**04:01 ^1^**	01:01 ^1^	03:01	**01:01**	05:01	**05:01**	02:01

^1^ SAT high-risk alleles; the same alleles detected in both patients are presented in bold.

## Data Availability

The source data are available on demand from the corresponding author.

## Abbreviations

ASIA	autoimmune/inflammatory syndrome induced by adjuvants
aTg	thyroglobulin antibodies
aTPO	thyroid peroxidase antibodies
CRP	C reactive protein
ECLIA	electrochemiluminescence immunoassay
ESR	erythrocyte sedimentation rate
FNAB	fine needle aspiration biopsy
FT3	free triiodothyronine
FT4	free thyroxine
GD	Graves’ disease
HLA	human leukocyte antigens
SAT	subacute thyroiditis
TRAb	thyrotropin receptor antibodies
TSH	thyrotropin
US	ultrasound
